# A mechanistic account of bodily resonance and implicit bias

**DOI:** 10.1016/j.cognition.2018.11.010

**Published:** 2019-03

**Authors:** Rachel L. Bedder, Daniel Bush, Domna Banakou, Tabitha Peck, Mel Slater, Neil Burgess

**Affiliations:** a UCL Institute of Cognitive Neuroscience, London, UK; b Max Planck UCL Centre for Computational Psychiatry and Ageing Research, London, UK; c UCL Queen Square Institute of Neurology, London, UK; d University of Barcelona, Event Lab, Department of Clinical Psychology and Psychobiology, Barcelona, Spain; e Mathematics and Computer Science Department, Davidson, USA; f UCL, Department of Computer Science, London, UK

**Keywords:** Virtual reality, Embodiment, Self-image, Implicit bias, Implicit association test, Neural network model

## Abstract

Implicit social biases play a critical role in shaping our attitudes towards other people. Such biases are thought to arise, in part, from a comparison between features of one’s own self-image and those of another agent, a process known as ‘bodily resonance’. Recent data have demonstrated that implicit bias can be remarkably plastic, being modulated by brief immersive virtual reality experiences that place participants in a virtual body with features of an out-group member. Here, we provide a mechanistic account of bodily resonance and implicit bias in terms of a putative self-image network that encodes associations between different features of an agent. When subsequently perceiving another agent, the output of this self-image network is proportional to the overlap between their respective features, providing an index of bodily resonance. By combining the self-image network with a drift diffusion model of decision making, we simulate performance on the implicit association test (IAT) and show that the model captures the ubiquitous implicit bias towards in-group members. We subsequently demonstrate that this implicit bias can be modulated by a simulated illusory body ownership experience, consistent with empirical data; and that the magnitude and plasticity of implicit bias correlates with self-esteem. Hence, we provide a simple mechanistic account of bodily resonance and implicit bias which could contribute to the development of interventions for reducing the negative evaluation of social out-groups.

## 1 Introduction

We tend to believe that the actions of ourselves and others are motivated by attitudes of which we are consciously aware and thus remain under cognitive control. However, our beliefs about others and, in particular, the social groups to which they belong play a demonstrable role in shaping behaviour. Biases regarding other people are often based on physical appearance, and likely driven by a comparison between cognitive representations of our own self-image and that of the other – a process termed ‘bodily resonance’ (Longo et al., 2009, Maister et al., 2015, Tsakiris, 2017). Perceived similarity between one’s self and another agent has been shown to modulate the ability to recognise emotions and bodily states in others. For example, perceived racial similarity has been shown to modulate both the increase in tactile acuity generated by watching another person being touched (Fini et al., 2013, Serino et al., 2009), and neural responses to observed painful experiences in others (Xu, Zuo, Wang, & Han, 2009). Similarly, when people interact with a virtual partner, the degree to which they mimic that partner’s gestures and posture – a sign of improved social harmony (Bargh & Chartrand, 1999) – is enhanced when their virtual skin colours are the same (Hasler, Spanlang, & Slater, 2017). This mirroring of observed bodily states in the self is thought to motivate pro-social behaviours where our ability to empathise can change our attitudes (Batson et al., 1997). Conversely, when implicit biases are negative, this can result in the systematic prevalence of unfavourable evaluations. A mechanistic understanding of implicit bias is therefore of critical importance both for the study of human behaviour and the functioning of society.

Interestingly, recent studies using immersive virtual reality (VR) to modulate bodily appearance have demonstrated that the cognitive representation of self-image is remarkably plastic. In these experiments, participants view a life-sized virtual human body from first-person perspective that visually substitutes for, and moves synchronously with, their own body. This creates a strong (illusory) perception of ownership over the virtual body. Changing the skin tone of the virtual body to that of a racial out-group for a short period (∼12 min) can subsequently reduce implicit bias against that out-group (Peck, Seinfeld, Aglioti, & Slater, 2013); and these reductions in implicit bias have been shown to persist for at least a week (Banakou, Hanumanthu, & Slater, 2016). Similarly, results obtained using the rubber hand illusion (Botvinick & Cohen, 1998) show that perceived ownership of a dark-skinned hand reduces implicit racial bias in light-skinned participants (Maister, Sebanz, Knoblich, & Tsakiris, 2013). This effect is not unique to racial out-groups, as self-identification with child-like attributes are enhanced after adults are embodied in a child’s body (Banakou, Groten, & Slater, 2013), and a reduction in age-related bias is also observed after younger participants are embodied in elderly bodies (Tajadura-Jiménez, Banakou, Bianchi-Berthouze, & Slater, 2017).

Here, we present a mechanistic account of bodily resonance that can be used to explain both the existence of implicit bias and the manner in which that implicit bias can be modified by an illusory body ownership experience. We postulate a distributed self-image network which contains groups of neurons that respond to specific features, and encodes associations between different features of an agent’s self-image. During subsequent perception of another agent, total output from the self-image network is proportional to the degree of overlap between that agent’s features and the encoded self-image, providing a mechanistic account of bodily resonance. Using output from the self-image network to drive a drift diffusion model of binary decision making, we can subsequently account for behavioural data obtained using a standard measure of implicit bias – the implicit association test (IAT; Greenwald, McGhee, & Schwartz, 1998). Moreover, additional learning of novel self-image features during simulated embodiment in a virtual body can modulate subsequent bias against specific out-groups. Finally, we show that both the magnitude and plasticity of implicit bias in these simulations is modulated by self-esteem, as predicted theoretically (Maister et al., 2015) and observed experimentally (Galinsky & Ku, 2004).

## 2 Results

### 2.1 The self-image network and bodily resonance

Our mechanistic account of bodily resonance centres on a hypothesised ‘self-image’ network in the brain comprised of neurons which selectively respond to various features that might constitute elements of a person’s self-image (i.e. gender, skin tone, hair colour, etc.). These neurons are activated by external sensory input whenever those features are perceived – whether they belong to the agent itself, or to any other agent or stimulus. Hence, each agent’s self-image network contains neurons which selectively respond to features that they themselves do not have, but have been observed in other agents (i.e. blonde haired agents will also have neurons in their self-image network that respond to brown hair, for example). Importantly, however, perception of the agent’s own bodily features enables associations to develop between active neural populations in the self-image network through Hebbian learning (Hasselmo, 2006, Hebb, 1949; see Section 4). We speculate that this privileged role for one’s own features might be driven by motor synchrony between those features and self-generated actions, potentially mediated by some neuromodulatory signal. The self-image network is therefore analogous to a standard model of auto-associative memory, encoding a single memory consisting of associations between different features of the agent’s self-image (Hopfield, 1982, Marr, 1971). Following encoding, the total firing rate output of the self-image network in response to external sensory input corresponding to perceived features provides a measure of bodily resonance, equivalent to the familiarity signal generated by auto-associative network models (Greve, Donaldson, & Van Rossum, 2010).

**Fig. 1:**
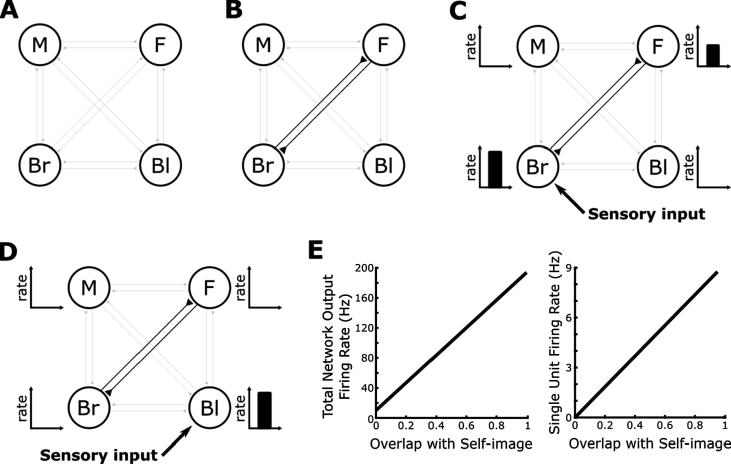
The Self-image Network Model of Bodily Resonance. (A) In this example, the self-image network is comprised of four neural sub-populations that respond to the perception of male (M), female (F), brown (Br) and blonde (Bl) haired features either in the agent itself or in other agents. (B) When the agent perceives its own features, a neuromodulatory signal allows synaptic connections between active sub-populations to be strengthened by a Hebbian learning rule. In this case, the simulated agent is a brown-haired female, such that the self-image network comes to encode strong associations between neurons encoding brown-haired and female features. (C, D) During subsequent perception of another agent, sensory input to sub-populations encoding the features of that agent generates additional activity in the network via recurrent synaptic connections if those features overlap with the encoded self-image. The total firing rate output of the self-image network, likely equivalent to the BOLD response observed in fMRI, can therefore be interpreted as a measure of bodily resonance. In this example, perception of a brown-haired agent produces activity in the sub-population encoding female features, while perception of a blonde-haired agent produces no additional activity in the self-image network. (E) Hence, the degree of overlap between the features perceived in another agent and the encoded self-image correlates with both the total output firing rate of the self-image network and the firing rate of any single unit encoding a feature that is part of the self-image, but not currently being perceived.

As an example, consider a simple self-image network consisting of *N* = 4 neurons that each encode a different potential feature: male, female, brown and blonde hair (Fig. 1A; noting that, without loss of generality, each feature would likely be encoded by a much larger population of neurons in the brain, and each agent’s self-image network would encode a much greater constellation of potential features). Let us assume that our simulated agent is a brown haired female, such that the self-image network will come to encode associations between neural populations encoding brown hair and female features (Fig. 1B). Once this self-image has been encoded, perceiving another agent with brown hair activates the corresponding neural population and generates recurrent currents which excite neurons encoding female features, boosting total network output (Fig. 1C). Conversely, perceiving another agent with blonde hair activates the corresponding neural population but generates no additional recurrent excitation, producing lower overall firing rates (Fig. 1D). Importantly, perceived features need not be physical – we might also postulate neural populations in the self-image network that encode more abstract characteristics, such as interest in a particular sport or a certain set of political beliefs (Serino et al., 2009), which will then also contribute to bodily resonance when observed in other agents.

More detailed simulations (see Section 4) demonstrate that, following the learning of associations between a constellation of arbitrary features belonging to a simulated agent, the total firing rate output of the self-image network in response to the subsequent perception of other agents correlates with the degree of overlap between perceived and encoded features (Fig. 1E). If we assume that this total firing rate output correlates with the BOLD response observed in fMRI, then this is equivalent to a parametric increase in the BOLD response of the self-image network according to the degree of overlap between the physical or abstract features of an observed agent and the self. Interestingly, as the additional network output is produced by recurrent excitation between neurons that encode those features, a measure of bodily resonance is also provided by the activity of any neuron or sub-population of neurons that form part of the encoded self-image. For example, bodily resonance between the brown-haired female agent and any other agent can be estimated by simply measuring the firing rate of neurons encoding either female or brown-haired features (Fig. 1E).

### 2.2 Modelling implicit bias

**Fig. 2:**
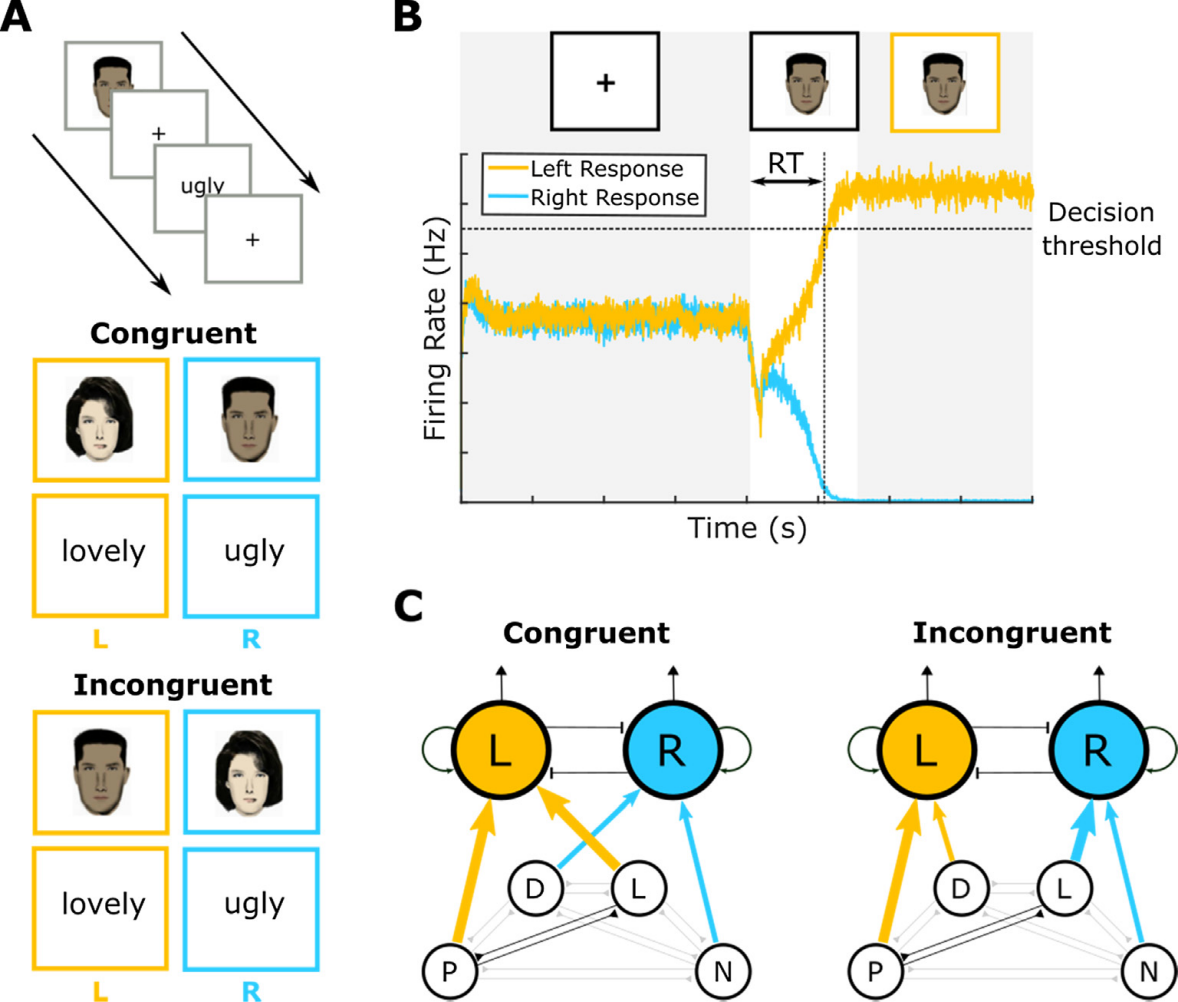
The Drift Diffusion Model of Behavioural Performance in the Implicit Association Test. (A) Each IAT trial consists of a fixation period followed by a stimulus that remains on screen until a response is made. Stimuli are either positive or negative ‘attributes’; in-group or out-group ‘targets’, and must be classified as quickly as possible by making a left (L) or right (R) key press. Reaction times (RTs) are compared between ‘congruent’ blocks, in which positive attributes and in-group stimuli require the same key press; and ‘incongruent’ blocks, in which positive attributes and out-group stimuli require the same key press, to produce an IAT score. (B) Behavioural performance on the IAT can be modelled using a drift diffusion model (DDM) in which two self-excitatory but mutually inhibitory neural populations coding for left and right motor outputs, respectively, noisily integrate external sensory evidence until the firing rate of one population reaches a pre-defined decision threshold. The time taken to reach the decision threshold produces an RT, while the winning population corresponds to the decision made (which may or may not correspond to the sensory evidence presented, i.e. be either correct or incorrect). (C) In our simulations, the sensory evidence provided to each DDM motor population in each IAT trial is determined by activity in the self-image network. Neurons coding for the IAT stimulus – Positive or Negative attributes and in- or out- group targets (Light- and Dark- skinned faces, respectively, in the example shown here) – receive a set level of external sensory input, while additional input to either motor response population arises from recurrent excitation within the self-image network (indicated by thicker coloured arrows). Note that connections from the self-image network to the DDM are flexibly reconfigured between congruent and incongruent blocks. (For interpretation of the references to colour in this figure legend, the reader is referred to the web version of this article.)

To make further comparisons between simulations of the self-image network and empirical data, however, we require the model to generate behavioural output. Measures of implicit bias are typically operationalised using the Implicit Association Test (IAT), a two alternative forced-choice task that requires participants to categorise binary sets of visual stimuli as quickly as possible by generating a specific motor response (Greenwald et al., 1998). Typically, two sets of stimuli are presented: one set of ‘attributes’, generally being positive and negative words; and one set of ‘targets’, generally being images of members of the social in-group and out-group of interest (male and female or light- and dark- skinned faces, for example). In alternate ‘congruent’ and ‘incongruent’ blocks, participants are instructed to generate the same motor response either for positive words and images of the in-group, and negative words and images of the out-group; or for positive words and images of the out-group, and negative words and images of the in-group, respectively (Fig. 2A). A comparison of reaction times between congruent and incongruent blocks subsequently provides a measure of implicit bias, with a positive IAT score indicating implicit positive bias towards the in-group (i.e. faster reaction times in congruent vs incongruent trials, supporting the idea of congruence between positive words and in-group features). Importantly, differences in reaction times during the IAT are largely unnoticed by participants and difficult to fake, making this task a suitable strategy for measuring implicit bias without the potential confound of social desirability (Greenwald et al., 2015, Hahn et al., 2014, Nosek et al., 2007, Oswald et al., 2013).

From a computational perspective, binary decision-making processes such as the IAT have been extensively modelled using drift-diffusion networks, in which two self-excitatory but mutually inhibitory neural populations noisily integrate sensory evidence for opposing motor responses until some firing rate threshold is reached and a decision is made (Bogacz et al., 2006, Klauer et al., 2007; Fig. 2B). Drift-diffusion models produce a reaction time for each decision in addition to a binary output regarding the decision made which may or may not correspond to the sensory evidence provided (i.e. be correct or incorrect). Greater sensory evidence for one motor response is accounted for by a greater level of external input to the corresponding neural population, causing the decision threshold to be reached more quickly and more reliably – i.e. with faster reaction times and lower error rates, as observed experimentally. Multiple iterations can subsequently be used to generate a reaction time distribution and error rate which can be directly compared with empirical data. Hence, we make use of a standard drift-diffusion model (Wong, Huk, Shadlen, & Wang, 2007) to examine the behavioural performance of our self-image network on a simulated IAT.

In standard binary decision making tasks, such as the moving dot paradigm (Huk & Shadlen, 2005), sensory evidence in favour of each motor output is experimentally defined (i.e. as the relative proportion of dots moving in each direction). In the case of the IAT, however, it is less clear how sensory evidence should be established for each visual stimulus (corresponding either to a positive or negative word, or an image of an in-group or out-group member, in congruent and incongruent blocks). We hypothesise that each stimulus category in the IAT (i.e. positive and negative words, in-group and out-group faces) is encoded by a sub-population of neurons in the self-image network which receive a constant level of external sensory input when stimuli from that category are presented. In addition, we assume that all neural populations encoding features that are mapped to a specific response in the IAT provide input, equivalent to sensory evidence, to the corresponding motor output population in the drift diffusion model.

Thus, during a block of congruent trials, we assume that transient connections are formed between neurons encoding positive words/features of the in-group and the same motor response population (although we do not explicitly model the formation of these connections). Equally, during a block of incongruent trials, we assume that connections are transiently formed between neurons encoding positive words/features of the in-group and different motor response populations (see Fig. 2C). Hence, in congruent trials (assuming that positive words are associated with in-group features in the self-image network), activity from a positive word or feature can spread to the corresponding motor response population via two routes, either directly or via recurrent connections within the self-image network, reducing reaction times. By contrast, on incongruent trials, recurrent activity in the self-image network spreading from a positive word to a feature of the in-group, for example, will then drive the opposing motor response population, increasing reaction times. Output from the self-image network to the drift diffusion model therefore provides a natural account of the IAT effect.

**Fig. 3:**
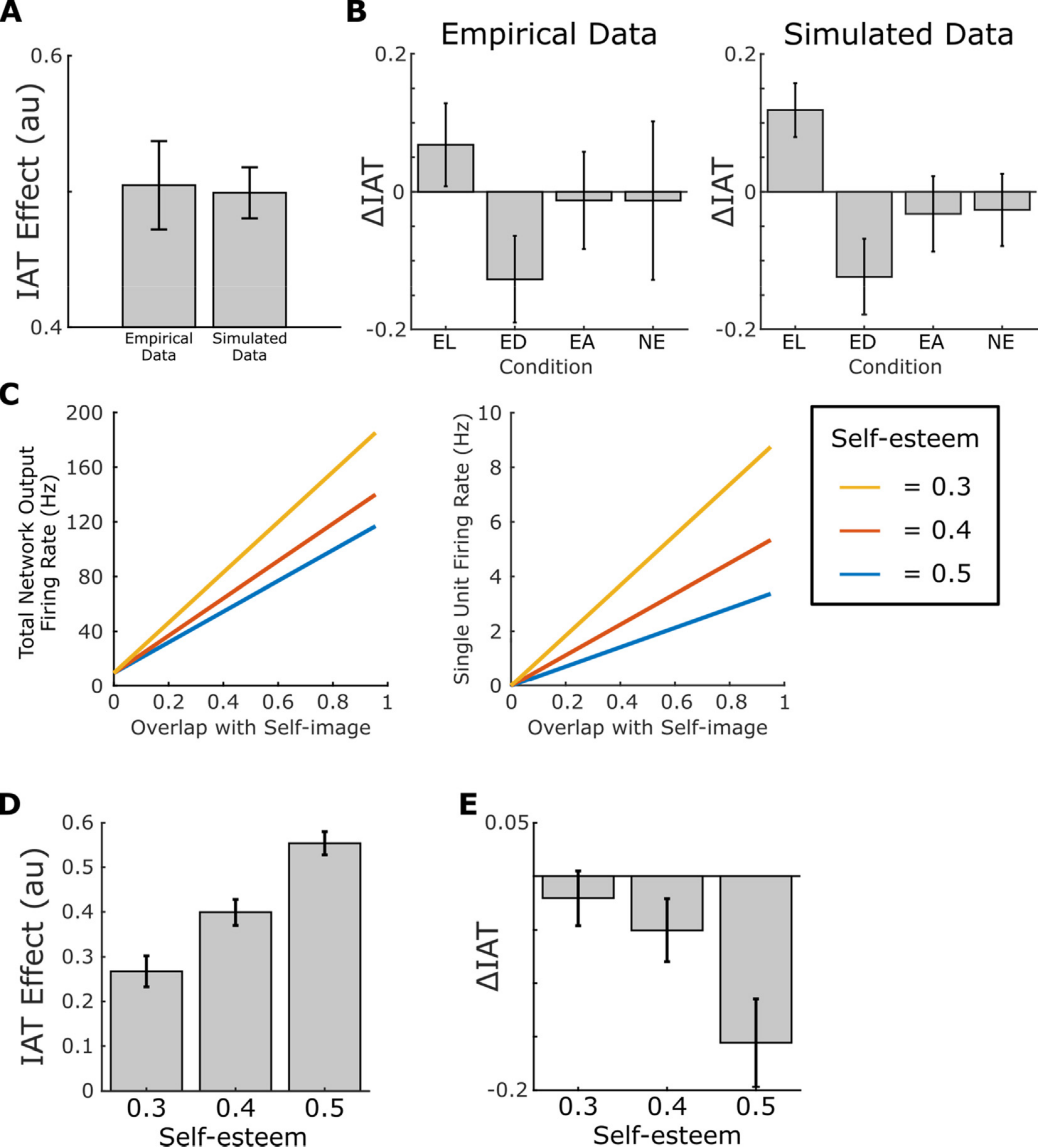
Empirical and Simulated Performance on the IAT. (A) Comparison of the IAT effect in empirical data from a population of sixty light-skinned females (see Peck et al., 2013 for details) and simulated data from a population of sixty light-skinned female agents, each of which shows significantly positive implicit bias towards other light-skinned agents (both p < 0.001). (B) Changes in the IAT effect (ΔIAT = IAT_post_ − IAT_pre_) produced by a short VR experience in real participants and simulated agents embodied in a light-skinned (EL); dark-skinned (ED); or alien (i.e. purple skinned) virtual body (EA); or who passively view another dark-skinned agent in the VR environment (NE). In both real and simulated data, embodiment in a dark-skinned virtual body generates a reduction in the IAT effect. Moreover, in the empirical data, embodiment in a light-skinned virtual body generates a small but non-significant increase in the IAT effect, which reaches significance in the simulated data. (C) The measure of bodily resonance produced by the self-image network is modulated by self-esteem: specifically, agents with lower self-esteem (i.e. reduced activity in neurons coding for positive features when the agent perceives its own body) exhibit lower overall bodily resonance (left), and lower firing rates in neurons coding for positive features (right), when observing other agents whose features overlap with their own. This reduction in bodily resonance corresponds to both (D) lower IAT scores and (E) less significant changes in IAT scores after being embodied in a dark skinned virtual body for agents with lower self-esteem. See 2.2, 2.4 for further details. (For interpretation of the references to colour in this figure legend, the reader is referred to the web version of this article.)

To illustrate this property, we simulate previously published experimental data obtained from a group of sixty light-skinned female participants performing a test of implicit racial bias (for further details, see Peck et al., 2013). Each simulated agent’s self-image network contains neurons that code for male and female, light- and dark- skinned features. In addition, we incorporate neural populations that code for positive and negative attributes, respectively, and which can be used to encode each agent’s measure of positive (or negative) self-esteem when they perceive their own image (see Section 4). First, each simulated agent learns about its own self-image by encoding associations between neural populations responding to light-skinned and female features and positive self-esteem. We subsequently simulate a racial IAT by presenting stimuli corresponding to positive and negative words, light- and dark- skinned features to the self-image network and using output from each population to drive motor response populations in the drift diffusion network (with light-skinned features and positive words driving the same motor population in congruent trials and competing populations in incongruent trials). This generates reaction times and error rates for blocks of congruent and incongruent trials which can be used to compute an IAT score for each simulated agent that can then be compared with empirical data. Our results demonstrate that the model produces significantly positive IAT scores across simulated agents (one-sample t(59) = 26.5, p < 0.001, 95% CI = [0.461 0.537], d = 3.42) analogous to that seen in real participants (one-sample t(59) = 15.5, p < 0.001, 95% CI = [0.44 0.57], d = 2.01; participant details can be found in Peck et al., 2013; see Fig. 3A). In fact, there is no difference between IAT scores generated in our simulations and those recorded experimentally (Kolmogorov-Smirnov test; D(118) = 0.20, p = 0.16).

In these simulations, the IAT effectively provides a mechanism with which to ‘read out’ the relative strength of implicit associations between various self-image features (i.e. gender, skin colour) and positive or negative attributes (arising from an agent’s self-esteem) stored in the self-image network. Strengthened recurrent connections between neural populations encoding those features produce a supralinear summation of input firing rates within those populations, which generates faster and more accurate responses in the drift-diffusion model when they provide input to the same motor response population. Alternatively, as described above, it is also possible to infer the degree of bodily resonance between each simulated agent and other agents they perceive by measuring simply the total firing rate output of the self-image network, likely equivalent to the observed BOLD response in fMRI. Accordingly, the total firing rate output of the self-image network in the light-skinned, female agents simulated above is much greater when perceiving light, as opposed to dark-, skinned faces, due to the greater overlap with their encoded self-image (cf. Fig. 1E).

### 2.3 Modulating implicit bias using immersive virtual reality

Having established that the self-image network can account for an empirically observed distribution of IAT scores, we next ask whether it can also replicate the observed changes in implicit bias generated by brief illusory body ownership experiences. Following (Peck et al., 2013), we divide our sixty light-skinned, female simulated agents between four VR conditions in which they are embodied in either a light (EL), dark (ED) or ‘alien’ (EA) skinned virtual body, or not embodied (NE) but passively observe a mirror reflection of a dark-skinned virtual body for the same period of time (see Peck et al., 2013 for further details). To simulate the VR experience, neurons in the self-image network that code for each feature of the virtual body are externally stimulated while learning proceeds for the embodied conditions, but not for the agents that passively view the virtual body (see Section 4). Each agent then completes a second, post-VR racial IAT as described above.

Our results demonstrate that simulated changes in implicit racial bias (ΔIAT) precipitated by the VR experience show the same pattern as empirical data, with implicit bias against dark-skinned individuals being selectively reduced in agents/participants that are embodied in a dark-skinned virtual body during the VR experience (Banakou et al., 2016, Peck et al., 2013; Fig. 3B). Specifically, we find a significant decrease in IAT scores in the ED condition (one-sample t(14) = −2.24, p < 0.05, 95% CI = [−0.24 to 0.0055], d = 0.58) which closely approximates that observed experimentally (one-sample t(14) = −2.01, p = 0.06, 95% CI = [−0.26 to 0.0083], d = 0.52; see Peck et al., 2013 for participant details). Similarly, we find a significant difference in ΔIAT scores between the EL and ED conditions (two-sample t(28) = 3.60, p < 0.005, 95% CI = [0.105–0.382], d = 0.57) which matches that observed experimentally (two-sample t(28) = 2.25, p < 0.05, 95% CI = [0.018–0.375], d = 0.40; see Peck et al., 2013 for further details). Importantly, although the original empirical study on which we base our simulations had relatively low power (power = 0.56 with α = 0.05; Peck et al., 2013), this result has since been replicated several times, suggesting that the observed effect is real (Banakou et al., 2016, Groom et al., 2009, Maister et al., 2013). In our simulations, the change in IAT scores arises from the novel associations between positive and dark-skinned self-image features generated during the VR experience, which produce increased bodily resonance with dark-skinned agents in the post-VR IAT, manifesting as reduced implicit bias against those agents.

Conversely, when simulated agents are embodied in an ‘alien’ virtual body, or passively view a dark-skinned virtual body, there is no significant change in IAT scores in either the simulated (one-sample t(14) = −0.51, p = 0.62 and t(14) = −0.60, p = 0.56, respectively) or empirical data (one-sample t(14) = −0.10, p = 0.92 and t(14) = −0.18, p = 0.86, respectively; see Peck et al., 2013 for further details), as no new associations are encoded in the self-image network. Interestingly, our results also replicate the increase in IAT scores – i.e. increase in bias towards light-skinned agents – generated by a VR experience in a light-skinned virtual body, which reaches significance in the simulations (t(14) = 3.06, p < 0.01, 95% CI = [0.0356–0.203], d = 0.79) but not in the empirical data (t(14) = 1.15, p = 0.27). This arises from the strengthening of associations between neurons in the self-image network encoding positive and light-skinned features during the VR experience, which subsequently produces increased bodily resonance with, and therefore bias towards, light-skinned faces in the post-VR IAT. Finally, we note the wide variance in ΔIAT scores observed in empirical data for the NE condition which, alongside the relatively small sample sizes, is likely to account for the absence of a difference in ΔIAT scores between the EL and NE (two-sample t(28) = 0.63; p = 0.54; 95% CI = [−0.184 to 0.347]; d = 0.23); ED and NE conditions (two-sample t(28) = −0.87; p = 0.39; 95% CI = [−0.383 to 0.154]; d = 0.32). This may be due, at least in part, to the low test-retest reliability of the IAT (see Section 3). Given larger sample sizes, however, the proposed mechanism predicts that ΔIAT scores for both the EL and ED conditions would differ significantly from those for the NE condition, in which no new associations are learned in the self-image network and therefore no change in bodily resonance is produced.

As described above, it is also possible to decode changes in implicit bias directly from changes in the total output firing rate (i.e. putative BOLD response) of the self-image network before and after the VR experience. In these simulations, the total firing rate output of the self-image network during the perception of dark-skinned faces is increased for those agents that are embodied in a dark-skinned body (ED), reflecting an increase in bodily resonance, but does not change for those agents that are embodied in a light-skinned body. This provides an empirically accessible measure of the change in implicit bias exhibited by these agents following the VR experience that is independent of the IAT.

### 2.4 Magnitude and plasticity of implicit bias is modulated by self-esteem

Having established that the self-image network model can replicate existing IAT data, we next ask whether it can be used to make any specific predictions for future behavioural experiments. For example, it has been hypothesised that self-esteem may modulate the IAT effect (Maister et al., 2015), while experimental data shows that reductions in implicit bias after imagining scenarios from the first-person perspective of an out-group member correlate positively with measures of self-esteem (Galinsky & Ku, 2004). To this end, one important property of the self-image network is that feature encoding need not be binary: the perception of fair hair, for example, might produce moderate firing rates in neural populations encoding both blonde and brown hair, such that a fair-haired agent will experience bodily resonance with other agents that have either blonde or brown hair. We can exploit this property to examine the effect of differences in self-esteem on the magnitude and plasticity of IAT scores (Banakou et al., 2016, Peck et al., 2013). When an agent is learning its own self-image, lower self-esteem can be modelled as lower firing rates in the neural population encoding positive features, producing lower synaptic weights between that population and those encoding other features in the self-image network. During the subsequent perception of other agents with features that match the encoded self-image, these reduced synaptic weights have a moderate effect on overall bodily resonance, but produce significantly lower firing rates in the neural population encoding positive features (Fig. 3C). In turn, this reduces the total output from the self-esteem network to the relevant motor output population in the drift diffusion model during congruent trials of the IAT, and hence the magnitude of IAT scores.

More detailed simulations (see Section 4) demonstrate that IAT scores are significantly modulated by self-esteem (One-way ANOVA, F(2,57) = 22.8, p < 0.001, η^2^ = 0.44; Fig. 3D), with those scores being significantly greater for agents with high self-esteem than those with both moderate (two-sample t(38) = 3.99, p < 0.001, 95% CI = [0.0762–0.233], d = 1.07) and low (two-sample t(38) = 6.59, p < 0.001, 95% CI = [0.198–0.374], d = 1.44) self-esteem; and those for agents with moderate self-esteem being significantly greater than those with low self-esteem (two-sample t(38) = 2.93, p < 0.01, 95% CI = [0.0406–0.223], d = 0.847). Moreover, during the VR experience, lower firing rates in the neural population encoding positive self-image features generate lower synaptic weights between neurons encoding dark-skinned and positive features, such that the change in IAT as a result of the VR experience is also reduced (Fig. 3E). Specifically, the change in IAT scores generated by the VR experience is significantly modulated by self-esteem (One-way ANOVA, F(2,57) = 4.75, p < 0.05, η^2^ = 0.143), with ΔIAT being significantly greater for agents with high self-esteem than those with both low (two-sample t(38) = 2.80, p < 0.01, 95% CI = [−0.233 to 0.0375], d = 0.82) and moderate self-esteem (two-sample t(38) = 2.09, p < 0.05, 95% CI = [−0.207 to 0.0033], d = 0.63; other p > 0.44). In summary, our model provides a neural level mechanism that account for the overall magnitude and plasticity of IAT scores across participants being modulated by measures of self-esteem, as hypothesised previously (Maister et al., 2015) and consistent with experimental data (Galinsky & Ku, 2004).

## 3 Discussion

We have presented a mechanistic account of bodily resonance and demonstrated that it can be used to explain both the existence of implicit bias and the plasticity of that bias following a brief illusory body ownership experience. Our account centres on a self-image network comprised of neurons that respond selectively to various features of a person’s self-image. Once this network has learned associations between the constellation of features belonging to the self, the perception of another agent with similar features generates recurrent excitation that boosts total network output, analogous to a measure of bodily resonance. By subsequently applying output from the self-image network to a drift-diffusion model of perceptual decision-making, we can simulate behavioural performance on an implicit association test (IAT) to quantify implicit bias. Consistent with empirical data, we demonstrate that simulated agents (which we assume to have positive features in their self-image) exhibit significantly positive IAT scores, and that further learning in the self-image network during an illusory body ownership experience can significantly modulate those IAT scores (Banakou et al., 2016, Peck et al., 2013). Finally, we show that both the magnitude and plasticity of implicit bias is modulated by self-esteem, as hypothesised previously (Maister et al., 2015) and supported by prior experimental findings (Galinsky & Ku, 2004).

The self-image network hypothesised here is maximally active when an agent perceives itself, due to the additional recurrent excitation produced by strengthened associations between neural populations encoding observed features. As such, the self-image network is likely to have been identified by neuroimaging studies seeking the neural correlates of self-recognition (Feinberg and Keenan, 2005, Keenan et al., 2001). Although it may be widely distributed, these studies suggest that the self-image network is right lateralised and centred on frontal (including inferior, medial and middle frontal gyri) and/or parietal (including the inferior parietal lobule, supramarginal gyrus and precuneus) cortices (see Devue and Bredart, 2011, Decety and Sommerville, 2003 for a review). Given that these neurons are active both when an agent perceives a specific feature in itself and in any other agent, it is interesting to note the overlap between these cortical regions and the mirror neuron system (Rizzolatti et al., 1999, Turjman, 2016). However, we emphasise that – unlike the mirror neuron system – the putative self-image network described here is insensitive to the perceived execution of any action; and must contain neurons that respond to features which have been perceived in other agents, but never formed part of the agent’s own self-image.

Interestingly, no significant change in implicit bias is observed experimentally following a VR experience in which participants are not embodied, but simply observe a virtual body that moves asynchronously with respect to their own actions (Peck et al., 2013). This effect is replicated by our model, given the assumption that additional learning in the self-image network requires a neuromodulatory signal contingent on motor synchrony between the perceived virtual body and simulated agent. Hence, this signal effectively encodes the level of body ownership felt by the simulated agent in the perceived virtual body. In the results presented here, we used binary levels of body ownership that either allowed or prevented any further learning in the self-image network during the simulated VR experience. However, there is no reason why this signal could not vary continuously according to the degree of body ownership felt by the simulated agent, modulating the degree of learning in the self-image network during any virtual embodiment experience and subsequently the magnitude of change in implicit bias following that experience. This is consistent with existing empirical data which demonstrates that proxy measures of presence (the feeling of physically being in the VR environment), such as the degree of nervousness felt by participants when other simulated agents approach them, correlate with the reduction in IAT scores observed after a VR experience (Hasler et al., 2017, Peck et al., 2013). In addition, while the model predicts no differences in the modulation of implicit bias produced by any specific paradigm, it may be that immersive VR paradigms generate a greater feeling of body ownership, and therefore precipitate more significant changes in implicit bias, than less immersive protocols such as the rubber hand or enfacement illusion (Sforza, Bufalari, Haggard, & Aglioti, 2010).

In addition to accounting for the reduction in implicit bias exhibited by light-skinned participants following embodiment in a dark-skinned virtual body, our model also replicates the increase in implicit bias sometimes exhibited by light-skinned participants following embodiment in a light-skinned virtual body (Banakou et al., 2016, Peck et al., 2013). This empirical finding might appear surprising, as it implies the further strengthening of associations between neurons in the self-image network encoding light-skinned and positive features during the VR experience, despite participants having had ample prior opportunity to encode these associations during their daily lives. It is not clear what precipitates this additional learning, but we hypothesise that the VR experience might be sufficiently novel to precipitate increased synaptic plasticity in the putative self-image network of those participants. Consistent with this view, experimental data indicates that the increase in implicit bias exhibited by light-skinned participants following embodiment in a light-skinned virtual body disappears with multiple exposures to the VR paradigm – presumably, as the experience becomes more familiar (Banakou et al., 2016). Conversely, the decrease in implicit bias exhibited by light-skinned participants following embodiment in a dark-skinned virtual body does not disappear following multiple exposures, suggesting either that the VR experience maintains its novelty, or that the synaptic connections between neurons encoding dark-skinned and positive features in those participants have not been saturated by a lifetime of exposure to their own self-image. In any case, the speed with which the encoded self-image is updated indicates that the network can flexibly respond to other rapid changes in an agent’s features which, importantly, need not be restricted to physical characteristics but also include abstract beliefs, opinions and allegiances.

### 3.1 Limitations

Despite this model qualitatively replicating a large body of experimental data, it also exhibits some limitations that merit further discussion. First, it must be emphasised that the output of the drift diffusion network is inherently noisy, leading to significant variability in the magnitude of implicit bias, and changes in implicit bias, across simulated agents. While this might provide a more realistic approximation of experimental data, it also highlights the need for larger samples – both in terms of participants, and the number of trials used in the IAT – for identifying pre-existing, and experimentally induced changes in, implicit bias. It is also important to note that, although the IAT has been widely used in studies of implicit bias, both its predictive and discriminant validity, internal and test-retest reliability have been questioned, with some meta-analyses suggesting that it is no better than explicit measures at predicting intergroup behaviour (Oswald et al., 2013). While this debate continues, it is important to note that the magnitude and plasticity of implicit bias described in these simulations can also be assayed by the total firing rate output of the self-image network, likely equivalent to the BOLD response observed in fMRI, and independent of the IAT.

Second, the model cannot account for all existing data relating to behaviourally induced manipulations of implicit bias – it is restricted to those that result from changes in self-image (i.e. that arise from bodily resonance). However, numerous experiments have demonstrated that interactions with members of an out-group can also modulate implicit bias, as described by the social contact hypothesis (Pettigrew and Tropp, 2006, Pettigrew, 1998). In addition, implicit biases are likely to reflect associations between specific groups and positive or negative valence learned slowly across a lifetime of social interaction (Banakou et al., 2016). Further work is needed to establish whether these data can also be accounted for by this model, or whether they imply an additional mechanism that contributes to overall implicit bias. Extending the model in this direction may also help to account for individual differences, which can only currently be accounted for in these simulations by differences in self-esteem. Similarly, experimental data demonstrate that the effects of manipulating self-image through VR experiences are not limited to changes in implicit bias, but can also modulate subsequent visual perception. For example, adults exhibit both an increased bias towards child-like attributes in an IAT and overestimate the size of physical objects within the environment following embodiment in virtual bodies corresponding to small children (Banakou et al., 2013, Tajadura-Jiménez et al., 2017). Again, further work is required to establish whether these effects can be accounted for by the framework described here. Finally, this simple model weights all features encoded by the self-image network with the same importance, such that implicit bias against out-group members defined by skin colour is identical to that against out-group members defined by hair colour, for example. However, a simple extension of the model could allow the impact of different features on the dynamics of the self-image network to be manipulated, by changing the size of the respective neural population(s) to modulate the amount of recurrent excitation produced and therefore the contribution to overall bodily resonance.

### 3.2 Predictions

Importantly, the model also makes several testable predictions for future empirical studies. First, it predicts parametric changes in the BOLD response generated by the self-image network according to the degree of overlap between an agent’s features and those perceived in other agents. Second, it predicts that neural populations within the self-image network will selectively respond to the perception of specific features, such as blonde hair, whether belonging to the self or any other agent. When those neurons encode features that do form part of the agent’s self-image, however, then they should also fire – at a lower rate – when the agent observes any other features that form part of its self-image, due to the strengthened recurrent connectivity between the respective neural populations. This property could also be probed using fMRI adaptation (Grill-Spector & Malach, 2001), where the network should generate a reduced BOLD signal when perceiving different features that form part of an agent’s self-image in quick succession, compared to perceiving different features that do not form part of an agent’s self-image. Finally, it is important to note that the reduction in implicit bias observed here is contingent on novel associations between out-group features and positive attributes being generated during the illusory body ownership experience. However, the model also predicts that implicit bias would be increased if those novel associations were instead formed between out-group features and negative attributes – if the participant experienced unpleasant or traumatic events during the body ownership illusion, for example. Hence, the model can also account for previous results that have demonstrated an increase in implicit racial bias following an immersive VR experience during which participants took part in a job interview (Groom et al., 2009).

### 3.3 Conclusion

In conclusion, we provide a mechanistic account of bodily resonance, associated implicit bias, and changes in that implicit bias induced by illusory body ownership experiences that modulate self-image. The understanding of implicit bias is of key importance to cognitive science, and also has the potential to contribute to the smooth functioning of society by identifying effective interventions that can reduce the negative evaluation of social out-groups. The results presented here, and in previous empirical studies, suggest that the use of virtual reality technology to allow embodiment in virtual bodies corresponding to members of the social out-group may represent a key strategy in pursuit of this goal.

## 4 Materials and methods

### 4.1 Self-image network model of bodily resonance

The self-image network consists of *N* neurons that are fully recurrently connected except for self-connections, analogous to an auto-associative network model of mnemonic function (Hopfield, 1982, Marr, 1971). To be parsimonious, we assume full recurrent connectivity, but network function should be qualitatively unaffected by reducing the level of recurrent connectivity between neurons. The firing rate output *r_i_* of neuron *i* is governed by a linear activation function which converts total input to a firing rate output, with a peak firing rate of *r_max_* = 10 Hz and a time constant of *τ_r_* = 10 ms (Eqs. (1a) and (1b)). A linear activation function is used for the sake of simplicity, and network function should be qualitatively unaffected by the use of other activation functions.

The total input to each neuron *I_tot, i_* is a sum of external sensory input *I_ext,i_* and recurrent synaptic currents *I_rec,i_* (Eq. (1c)). Each neuron codes for a specific self-image feature, and a constant external input of *I_ext,i_* = 0.5 is applied to that neuron whenever the simulated agent perceives either itself or another agent with that feature. Recurrent synaptic currents are equal to the product of synaptic weights *w_ij_* and firing rates of connected neurons *r_j_* (Eq. (1d)). All firing rates *r_i_* and synaptic connections *w_ij_* within the network are initially set to zero.

Changes in the strength of synaptic connections between pre-synaptic neuron *i* and post-synaptic neuron *j* are governed by a Hebbian learning rule (Hebb, 1949), i.e. proportional to the product of pre- and post-synaptic firing rates and a learning rate *k* = 2 × 10^−5^ (Eq. (1e)). In addition, we postulate an abstract neuromodulatory signal *e* that differentiates between periods of encoding (*e* = 1), during which the self-image network encodes associations between different features that the agent perceives as belonging to itself; and retrieval (*e* = 0), during which the self-image network produces a measure of bodily resonance between the encoded self-image and a set of features currently perceived in another agent (Hasselmo, 2006). This neuromodulatory signal inversely modulates the magnitude of synaptic plasticity (for encoding) and recurrent synaptic currents (for retrieval; see Eqs. (1b) and (1d)).

(1a) $$I_{\mathrm{tot},i}=\left\{\begin{array}{c} I_{\mathrm{tot},i}\text{f}\text{o}\text{r}I_{\mathrm{tot},i}\leq r_{\max}\\ r_{\max}\text{f}\text{o}\text{r}I_{\mathrm{tot},i}>r_{\max} \end{array}\right)$$

(1b) $$\tau_{r}\frac{dr_{i}}{\mathrm{dt}}=-r_{i}+I_{\mathrm{tot},i}$$

(1c) $$I_{\mathrm{tot},i}=I_{\mathrm{ext},i}+I_{\mathrm{rec},i}$$

(1d) $$I_{\mathrm{rec},i}=\left(1-e\right)\sum_{n=1}^{j}r_{j}w_{\mathrm{ij}}$$

(1e) $$w_{\mathrm{ij}}=w_{\mathrm{ij}}+ekr_{i}r_{j}$$

To generate the data presented in Fig. 1E, we simulate one agent with a self-image network consisting of *N* = 40 neurons that each code for an arbitrary feature. During an initial learning phase lasting 10 s, the agent perceives its own features, such that twenty of the neurons in the self-image network are externally stimulated with a constant current of *I_ext_* = 0.5. During this period, the neuromodulatory signal is set to *e* = 1 to allow learning to proceed in the self-image network (Fig. 1B). Following this learning phase, we examine the level of bodily resonance produced by the self-image network by externally stimulating twenty neurons in the self-image network with the same level of external stimulation and recording output firing rates after a period of 1 s. During this period, the neuromodulatory signal is set to *e* = 0, reflecting the fact that the simulated agent is now perceiving another agent, such that no learning proceeds, but allowing recurrent synaptic currents to generate additional activity in the network (Fig. 1C and D). In each of twenty simulations, the degree of overlap between encoded self-image and perceived features of the other agent is systematically varied. Importantly, the total level of external stimulation remains constant across each of these simulations – differences in total firing rate output of the network being generated by changes in the magnitude of recurrent synaptic currents only.

### 4.2 Drift diffusion model of the implicit association test

In drift diffusion models of two alternative forced choice tasks, two competing neural populations encode the cumulative level of evidence for left and right motor responses, respectively. Once activity in one or the other population reaches a pre-defined firing rate threshold, then the decision is made and the corresponding motor response is enacted. Here, we make use of a reduced two-variable drift diffusion model previously described by Wong et al., 2007, Wong and Wang, 2006. This choice is motivated by computational efficiency, and the results reported should be qualitatively unaffected by the use of other drift diffusion network models.

In this model, the firing rate of each population *r_i_* (where *i* = L, R) is dictated by the relationship between synaptic current input *I_i_* and spike output for an integrate and fire neuron (Abbott & Chance, 2005), with parameter values of *a* = 270 Hz/nA, *b* = 108 Hz and *d* = 0.154 s (Eq. (2a)). The total synaptic input to each population is a sum of stimulus driven sensory-input *I_stim_*, input from fixation cross *I_fix_*, background synaptic inputs *I_noise_* and the excitatory and inhibitory synaptic couplings both between and within populations, *J_ii_* = 0.3275 nA and *J_ij_* = 0.1137 nA respectively (Eq. (2b)). NMDA currents in each population *S_i_* are dictated by the instantaneous firing rate in that population modulated by a gain parameter *γ* = 0.641, and decay with a time constant of *τ_s_* = 60 ms (Eq. (2c)). Each population receives background synaptic inputs, representing inherent noise in the network, with a mean value of *I_0_* = 0.3297 nA and a white noise component *η_i_(t)* with an amplitude of *σ_noise_* = 0.009 nA that is filtered by a synaptic time constant of *τ_noise_* = 2 ms (Eq. (2d)).

(2a) $$r_{i}=f\left(I_{i}\right)=\frac{aI_{i}-b}{(1-\exp\left[-d\left(aI_{i}-b\right)\right]}$$

(2b) $$I_{i,total}=J_{\mathrm{ii}}S_{i}-J_{\mathrm{ij}}S_{j}+I_{\mathrm{stim},i}+I_{\mathrm{fix}}+I_{\mathrm{noise},i}$$

(2c) $$\frac{dS_{i}}{\mathrm{dt}}=-\frac{S_{i}}{\tau_{s}}+\left(1-S_{i}\right)\gamma f\left(I_{i}\right)$$

(2d) $$\tau_{noise}\frac{dI_{\mathrm{noise}}\left(t\right)}{\mathrm{dt}}=-\left(I_{\mathrm{noise}},i\left(t\right)-I_{0}\right)+\eta_{i}\left(t\right){\sqrt{\tau }}_{\mathrm{noise}}\sigma_{noise}$$

Each simulated IAT trial begins with the presentation of a fixation cross at *t_fix_*, which is associated with a small excitatory input *I_fix_* to each population in the drift diffusion mode (Eq. (3a)). This input, which is modulated by a gain factor of *J_A,ext_* = 1.1 × 10^−3^ nA/Hz and exhibits short-term adaptation with a time constant of *τ_ad_* = 40 ms, serves to bring activity in two motor populations to a stable equilibria in which they each fire at a moderate rate (Fig. 2B). The amplitude of this current is reduced when the visual stimulus appears at *t_stim_*, and supplemented by differential levels of input to each population *I_stim_* proportional to the level of sensory evidence *s*′ available in favour of one decision or the other, modulated by a gain factor *f* = 0.45, with a mean value of µ_0_ = 30 Hz (Eq. (3b)).

(3a) $$I_{\mathrm{fix}}=\left\{\begin{array}{c} J_{A,ext}\left(50+100exp\left[-\left(t-t_{\mathrm{fix}}\right)/\tau_{\mathrm{ad}}\right]\right)\text{f}\text{o}\text{r}\;t_{\mathrm{fix}}\leq t<t_{\mathrm{stim}}\\ J_{A,ext}\left(6+44exp\left[-\left(t-t_{\mathrm{stim}}\right)/\tau_{\mathrm{ad}}\right]\right)\text{f}\text{o}\text{r t}>t_{\mathrm{stim}} \end{array}\right)$$

(3b) $$I_{\mathrm{stim},i}=J_{A,ext}\mu_{0}\left(1+fs^{\text{'}}\right)$$

In our model, the level of sensory evidence in each IAT trial is derived directly from activity in the self-image network. Specifically, neurons in the self-image network coding for the visual stimulus are externally stimulated with a constant current of *I_ext_* = 0.5 while the neuromodulatory signal is set to *e* = 0 to allow recurrent synaptic currents but prevent further learning. Once firing rates in the network reach an equilibrium state, we sample the total firing rate output in neural populations coding for features that are associated with each motor response in the IAT (Fig. 2C). The total level of sensory evidence *s*′ is subsequently proportional to the difference in total firing rate input to each motor population, excluding the firing rate activity generated by external stimulation *I_ext_* (Eq. (4)). Importantly, we assume that connections from the self-image network to the drift diffusion network are flexibly reconfigured between congruent and incongruent blocks, but do not explicitly model these changes in connectivity. Once the firing rate in either motor response population in the DDM reaches a firing rate threshold of *r_thresh_* = 55 Hz, the reaction time and decision made are recorded and the next trial begins.

(4) $$s^{\text{'}}=\left(\sum r_{L}-\sum r_{R}\right)-I_{\mathrm{ext}}+0.05$$

The structure of the simulated IAT is consistent with empirical studies (Banakou et al., 2016, Peck et al., 2013). Specifically, each participant/simulated agent performs two congruent and incongruent blocks, each consisting of 48 trials, with the order of blocks counterbalanced across participants. Trials with reaction times of >10 s and participants who have >10% of trials with reaction times <300 ms are excluded. In addition, reaction times for error trials are replaced by the mean reaction time for that block plus 600 ms. The IAT score is then given by the mean RT across congruent trials subtracted from the mean RT across incongruent trials, normalised by the standard deviation of all trials prior to any error corrections (Eq. (5)).

(5) $$IATscore=\frac{\bar{{\mathrm{RT}}_{\mathrm{incon}}}-\bar{RT_{\mathrm{con}}}}{{STD}_{all}}$$

To generate the data presented in Fig. 3A, we simulate sixty agents with self-image networks consisting of *N* = 6 neurons that code for male, female, light skinned, dark skinned, positive and negative features, respectively. We assume that each neuron encodes a single feature only, rather than a conjunction of multiple features, for the sake of simplicity only – the conjunctive coding of features by neurons in the self-image network would have no qualitative effect on the results presented. During an initial learning phase lasting 10 s, each agent perceives its own features (female, light skinned and positive), such that the corresponding neurons in the self-image network are externally stimulated with a constant current of *I_ext_* = 0.5. During this period, the neuromodulatory signal is set to *e* = 1 to allow learning to proceed in the self-image network.

We subsequently compute the level of bodily resonance produced by each agent’s self-image network in response to each IAT stimulus by externally stimulating the corresponding neuron in the self-image network (i.e. that coding for light skinned, dark skinned, positive or negative features) with the same level of external stimulation while the neuromodulatory signal is set to *e* = 0, recording output firing rates after a period of 1 s, and computing the level of sensory evidence separately for congruent and incongruent trials according to Eq. (4). These sensory evidence values are then used to replicate the structure of the IAT task described above, and reaction times and error rates across congruent and incongruent trials used to compute an IAT score for each agent according to Eq. (5).

To generate the data presented in Fig. 3B, we simulate an additional 2 s learning period during which neurons in the self-image network coding for a set of condition specific features are externally stimulated with a constant current of *I_ext_* = 0.5. For agents in the EL condition, these are neurons coding for female, light skinned and positive features; and for agents in the ED and NE conditions, these are neurons coding for female, dark skinned and positive features. Agents in the EA condition are randomly assigned to perceive the purple skin tone as either light or dark, and then neurons coding for female and positive features are stimulated along with those for the randomly selected skin tone. For the embodied conditions (EL, ED, and EA), the neuromodulatory signal is set to *e* = 1, reflecting the fact that the virtual body moves synchronously with their own; while for the NE condition, the neuromodulatory signal is set to *e* = 0, reflecting the fact that the agent is perceiving another agent. After this additional learning period, sensory evidence for each IAT stimulus is computed separately for congruent and incongruent trials, and IAT scores are generated as described above.

Finally, to examine changes in the dynamics of the self-image network when the self-esteem of simulated agents is systematically varied (Fig. 3C–E), we simply modulate the level of external stimulation to neurons in the self-image network coding for positive features during the initial 10 s and subsequent 2 s learning periods, such that those neurons fire at a lower rate while the agent encodes associations between its self-image features. Specifically, we vary the level of constant current to neurons encoding positive features between *I_ext_* = 0.3 and *I_ext_* = 0.5 while all other neurons encoding features of the simulated agent receive a constant current input of *I_ext_* = 0.5, as described above.
